# Technology-Based Rehabilitation to Improve Communication after Acquired Brain Injury

**DOI:** 10.3389/fnins.2017.00382

**Published:** 2017-07-28

**Authors:** Carrie A. Des Roches, Swathi Kiran

**Affiliations:** Aphasia Research Laboratory, Speech, Language, and Hearing Sciences, Sargent College, Boston University Boston, MA, United States

**Keywords:** aphasia, stroke, acquired brain injury, technology, rehabilitation

## Abstract

The utilization of technology has allowed for several advances in aphasia rehabilitation for individuals with acquired brain injury. Thirty-one previous studies that provide technology-based language or language and cognitive rehabilitation are examined in terms of the domains addressed, the types of treatments that were provided, details about the methods and the results, including which types of outcomes are reported. From this, we address questions about how different aspects of the delivery of treatment can influence rehabilitation outcomes, such as whether the treatment was standardized or tailored, whether the participants were prescribed homework or not, and whether intensity was varied. Results differed by these aspects of treatment delivery but ultimately the studies demonstrated consistent improvement on various outcome measures. With these aspects of technology-based treatment in mind, the ultimate goal of personalized rehabilitation is discussed.

## Introduction

It is estimated that ~100,000 individuals acquire aphasia each year in industrial countries (eso-stroke.org). Even though stroke-induced aphasia is more debilitating than other disabilities (Lam and Wodchis, 2010; Worrall et al., 2011), individuals with aphasia do not always receive the rehabilitation that they require. There are several studies that have demonstrated the beneficial effects of rehabilitation in the acute stages after stroke (Laska et al., 2011; Godecke et al., 2012) as well as in the chronic stages (Kiran and Sandberg, 2011; Allen et al., 2012). Critically, a recent review of treatment studies in chronic post-stroke individuals found that treatment outcomes for individuals in the chronic phase (6 months or longer post stroke) was quite robust, questioning the premise that chronic post-stroke individuals do not benefit from rehabilitation (Allen et al., 2012; Teasell et al., 2012). These and other studies highlight the importance of providing sustained rehabilitation to acute and chronic patients. Even though these individuals clearly require long-term rehabilitation it is not always provided due to practical and financial constraints. Specifically, rehabilitation currently involves high levels of clinician involvement, limiting the number of individuals they can work with at a given period of time and is, therefore, not cost-effective (Palmer et al., 2012; Wenke et al., 2014). Further, due to limited coverage in health care plans for speech and cognitive rehabilitation, many individuals receive limited or no support to continue rehabilitation or maintain the progress they made in treatment. Some geographical areas have a prominent stroke community that provides support and continued rehabilitation in group and individual settings for individuals who are motivated to search for such programs. However, individuals living in remote areas often experience difficulty receiving services, even if they are motivated to continue working on their recovery. Advancements of technology-based rehabilitation for individuals with acquired brain injury has provided a potential solution to the issues faced when seeking rehabilitation.

A second potential benefit of technology-based rehabilitation is that the same level of involvement by clinicians can provide patients with a greater, **intensity of aphasia rehabilitation**, which is most often manipulated by increasing the number of sessions (Laganaro et al., 2006). Additionally, the use of technology to track the number of items completed in a session can be an important tool for clinicians, since it may have an effect on outcomes (Harnish et al., 2014). Indeed, technology can supplement traditional methods of rehabilitation by providing the opportunity for continued rehabilitation as per the convenience of the individual. Importantly, a review found that greater intensity is a positive prognosticator for overall long term recovery (Bhogal et al., 2003a), providing further evidence for the beneficial effects of rehabilitation. The use of technology-based rehabilitation is one way to provide greater intensity and, if proven effective, can be an important tool for clinicians to improve rehabilitation outcomes.

KEY CONCEPT 1 **Intensity of aphasia rehabilitation**

Refers to the time per week spent on treatment throughout the duration of the study. This term reflects how concentrated the treatment is during a given week of the study that an individual receives. This is a main advantage of technology-based treatment, which is allowing for greater levels of intensity.

Recently, reviews have examined technology-based rehabilitation, both for cognitive deficits (Bogdanova et al., 2016; Sigmundsdottir et al., 2016) and language deficits (Lee and Cherney, 2016; Zheng et al., 2016) and have found preliminary evidence for the effectiveness of this method of rehabilitation. Importantly, the reviews highlight the need for more research due to vast differences in methodological designs, thereby, limiting the breadth of conclusions that can be drawn about the efficacy of such approaches. Additionally, these reviews have been narrow in their scope of the studies reviewed; i.e., most have focused on specific types of interventions. For instance, Zheng et al. (2016) examined only studies that had a control group of either no treatment or clinician delivered treatment and therefore only reviewed seven studies, while Lee and Cherney (2016) provided a narrative review of a selected sample of previous studies in order to provide an overview of the variety of technology-based treatments available. In summary, by extrapolating the results from technology-based language and cognitive rehabilitation approaches, we can begin to examine the various factors that influence outcomes including the method of treatment delivery, the types of **outcome measures** utilized, and the frequency and intensity of treatment practice. Further, we can also begin to address questions about how different aspects of the delivery of treatment can influence rehabilitation outcomes.

KEY CONCEPT 2 **Outcome measures**

**Figure 1:**
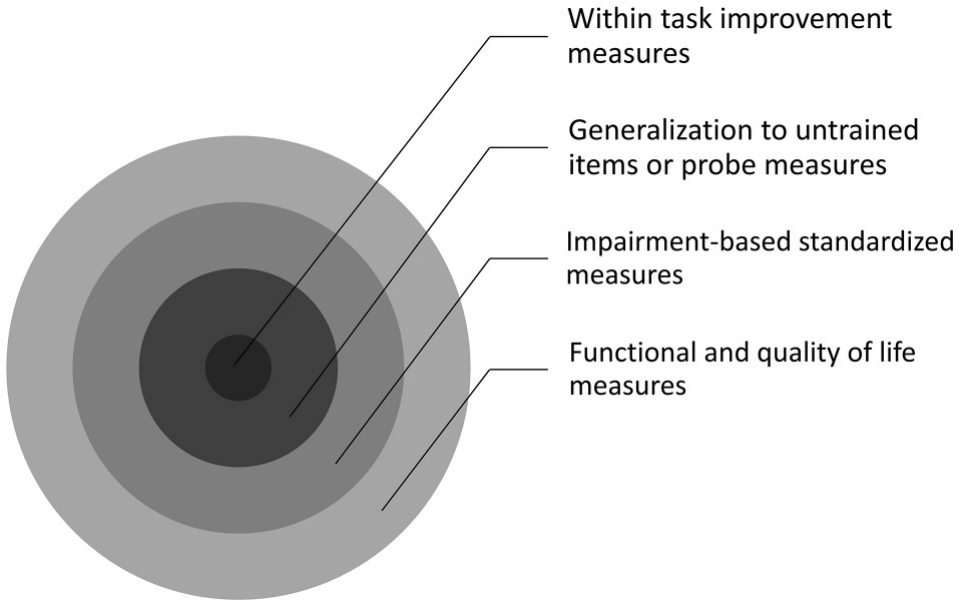
Schematic depicting the different types of outcomes that are examined across previous technology-based treatment studies in Tables 1, 2. Within task improvement measures are the closest to what is being treated, while generalization to untrained items or probe measures goes a step farther away from what is trained, followed by impairment-based standardized measures and then functional and quality of life measures.

Refers to how improvement is measured. There are various types of outcome measures that capture differing levels of improvement (see Figure 1 for more detailed information).

## Previous technology-based rehabilitation studies

There have been many studies with wide-ranging experimental designs that have investigated the effectiveness of technology-based language and cognitive rehabilitation with individuals with acquired brain injury (see Table 1). The treatment provided in these studies has varied extensively, as some provide language rehabilitation in either a single domain or multiple domains, while others provide both language and cognitive rehabilitation. In this review, computer-based cognitive rehabilitation for traumatic brain injury will not be included, as two recent meta-reviews have provided extensive examinations of such intervention studies (Bogdanova et al., 2016; Sigmundsdottir et al., 2016). This review will instead focus on language based intervention utilizing technology-based programs in individuals with stroke-induced aphasia. A literature search in 2016 with a keyword search (e.g., technology treatment/rehabiltiation aphasia, iPad treatment/rehabilitation aphasia, computer treatment/rehabilitation ahpasia, etc.) utilizing several databases (PubMed, speechBITE, and Google Scholar) found 31 studies that examined technology-based rehabilitation in either language or language and cognitive domains. The remaining studies were excluded if they (i) did not include treatment programs, (ii) were single case studies with fewer than three participants^1^, (iii) provided different treatments for each individual, (iv) included a primary population of individuals with primary progressive aphasia or dementia, (v) the technology was only used as an augmentative/alternative communication device or as an assessment tool, or (vi) only reported subsets of the full data from studies that were published elsewhere.

**Table 1:** Information about previous technology-based rehabilitation studies that have provided language rehabilitation to individuals with aphasia.

	**Study**	**Participants**			**Treatment**		**Main results**
		N, age, MPO^*^	**Etiology and severity**	**Control group**	**Type**	**Duration and intensity**	
Language, single domain, naming	Aftonomos et al., 1997	N:23, age:64.3, MPO:46.3 (all chronic)	Stroke^*^ **(mostly)** Mixed levels of severity and aphasia type	**No**	**Lingraphica** Interactive lexical items in the major linguistic categories that appear in a field of semantically related items; works on word retrieval on multiple levels	Mean duration:16.8 weeks (varied), intensity mean:1.99 sessions per week in clinic, variable intensity decided by patient for homework	All standardized tests (WAB^*^, BNT^*^, BDAE^*^) showed gains for most patients
	Fink et al., 2002	N:6, age:60.5, MPO:49.2 (all chronic)	**Stroke** Mixed levels of severity and aphasia type	**No**, two equal groups (full clinician guidance vs. partial guidance)	**MossTalk Words** Cued naming	4 weeks or until criterion, 3 times per week; variable intensity decided by patient for independent practice in partial guidance group	Both groups showed gains on trained words (as measured by PNT^*^), gains on PRT^*^ for one clinician-guided and on PORT^*^ for two partial-guided patients
	Raymer et al., 2006	N:5, age:70.8, MPO:92 (2 were subacute, 3 were chronic)	**Stroke** Mixed levels of severity and aphasia type	**No**, two levels of intensity (crossed design)	**MossTalk** Multimode matching exercises	Each training phase: 12 sessions, lower intensity: 1–2 times per week, higher intensity: 3–4 times per week	All patients improved in trained items, more in higher intensity phase, one patient showed gains on WAB AQ^*^ and BNT
	Ramsberger and Marie, 2007	N:4, age:67.5, MPO:31.5 (all chronic)	**Stroke** Mixed levels of severity and aphasia type,	**No**, two levels of intensity (crossed design)	**MossTalk Words** Self-cued naming with partial clinician guidance	15–20 sessions per word list Lower intensity: 2 times per week Higher intensity: 5 times per week	Three patients showed gains in naming, regardless of intensity
	Doesborgh et al., 2004	N:18, age:62 (EG^*^), 65 (CG^*^), MPO:13 (EG), 13 (CG) (all chronic)	**Stroke** Moderate to severe, global aphasia excluded	**Yes** No treatment (N:10)	**Multicue** Self-cued naming	2 months, 2-3 times per week	EG showed gains on BNT, but no between group differences
	Loverso et al., 1992	N:21 (all chronic)	**Stroke** Mixed levels of severity and aphasia type	**No**, alternating between clinician and computer/clinician delivered	Cueing Verb Treatment, incorporates both semantic and syntactic structures	Until reached criterion, varied by patient	Clinician was more effective than computer for treatment improvement, gains for 18/21 patients on PICA^*^
	Bruce and Howard, 1987	N:5, age:47.9, MPO:~28.32 (all chronic)	**Stroke** Broca's aphasia	**No**	Microcomputer as an aid to generate phonemic cues	5 sessions total	All patients improved, four were better on trained words and untrained words, four were better at indicating first letter of names in trained set than untrained
	Fridriksson et al., 2009	N:10, age:59.2, MPO:85.3 (all chronic)	**Stroke** Non-fluent aphasia, mixed levels of severity	**No**, alternating between two treatment conditions	Picture/word matching tasks: AV treatment: audio-visual speech stimuli, AO treatment: audio only speech stimuli	3–6 weeks, 5 days a week for 30 min	Naming of trained items and PNT improved after AV but not AO, no between group differences
	Harnish et al., 2014	N:8, age:56.5, MPO:52.5 (all chronic)	**Stroke** Mixed levels of severity and aphasia type	**No**	Computerized confrontation naming with multiple levels of cues	2 weeks, 4 days a week for 60 min	All patients showed an improvement on trained items during treatment after 1–3 sessions, with all patients showing significant ES^*^ on trained items varying from small to large
	Kurland et al., 2014	N:5 (8 completed but only 3 in data analysis), age:67.4, MPO:31.9 (all chronic)	**Stroke** Mixed levels of severity and aphasia type	**No**	Self-managed iPad task for maintaining and improving object and verb naming with cues	6 months, variable intensity decided by patient, average practice: 18 min per day	All patients maintained previous gains and gained new trained words, BDAE and BNT scores equal to or better than baseline
	Woolf et al., 2016	N:20, age:53–67 MPO:20.2–53.4 (depends on cohort) (all chronic)	**Stroke**	**Yes** 4 groups: Remote treatment from university or clinic, face-to-face, and attention control group (N:5 per group)	Remote treatment delivered via FaceTime, homework practice: PowerPoint with cues built in to improve spoken word production; attention CG: conversation sessions only	4 weeks, 2 times per week, variable intensity decided by patient for homework	Trained items improved in all treatment groups but not CG, clinic EG scored higher than other two groups and face-to-face scored higher than univ.EG; no change in POWERS^*^ for any group
Language, Single domain, Reading	Katz and Wertz, 1992	N:43, age:59.5–65.6, MPO:45.2–81.84 (depends on cohort) (all chronic)	**Stroke** Between 15th and 90th overall percentile on PICA	**Yes** 2 CGs: Computerized non-verbal games and cognitive tasks, (stim EG (N:15)); No treatment (N:15)	Computerized reading treatment that involved visual matching and reading comprehension tasks; with adjustable task difficulty	6 months, 3 h per week	Reading EG showed gains on trained items, PICA and WAB AQ; with more gains on PICA than other groups
	Katz and Wertz, 1997	N:55, age:60's, MPO:64.8–102 (depends on cohort) (all chronic)	**Stroke** Between 15th and 90th overall percentile on PICA	**Yes** 2 CGs: Computerized non-verbal games and cognitive tasks, (stim EG (N:19)); No treatment (N:15)	Computerized reading treatment that involved visual matching and reading comprehension tasks; with adjustable task difficulty	26 weeks, 3 h per week	Reading EG showed more gains than other groups on PICA and WAB Repetition and AQ
	Cherney, 2010	N:25, age:56.6 (EG), 61.1 (SLP), MPO:66.7 (EG), 41.3 (SLP) (all chronic)	**Stroke** Non-fluent aphasia (global excluded)	**Yes** Computer vs. clinician delivered (N:11), no treatment period for both groups	**ORLA** (oral reading for language in aphasia) on computer: reading aloud sentences and paragraphs with computer-voice and then independently	24 sessions, 1–3 times per week	Groups had equal gains in WAB-R^*^ AQ and discourse measures compared to no treatment; no between group difference
Language, Single domain, Sentence Processing and Production	Cherney and Halper, 2008	N:3, age:64, MPO:36 (all chronic)	**Stroke** Two non-fluent, one fluent	**No**	**AphasiaScripts** Virtual therapist for script training with diminishing cues	3 weeks, variable intensity decided by patient	2 had gains on trained scripts, 1 improved on WAB AQ and CETI^*^ and 1 improved on QCL^*^
	Manheim et al., 2009	N:20, age:54.8, MPO:53.0 (all chronic)	**Stroke** Mixed levels of severity and aphasia type	**No**, though delayed treatment for all participants	**AphasiaScripts** Virtual therapist for script training with diminishing cues	9 weeks, variable intensity decided by patient, average: 44 h total	CD^*^ subscale of BOSS^*^ had no change during delay period and gains during treatment period
	Cherney et al., 2014	N:8, age:52, MPO:26.5 (all chronic)	**Stroke** Mixed levels of severity and aphasia type	**No**, though comparing two conditions of cueing	**AphasiaScripts** Virtual therapist for script training with diminishing cues High cue: normal program, Low cue: written cue until 3 independent trails	3 weeks per condition with 3 weeks in between	Trained scripts increased in accuracy and rate; conditions were no different in results
	Kalinyak-Fliszar et al., 2015	N:4, age:50, MPO:55.5 (all chronic)	**Stroke** Mixed levels of severity and aphasia type (no global)	**No**, comparing virtual clinician (VC) to human clinician (HC) (crossed design)	Computer-delivered scripts with virtual clinician, and real clinician controlling computer output	1 week, 4 sessions of 30–40 min each	Trained script results varied by patient; two patients made gains on discourse narratives
	Thompson et al., 2010	N:12, age:49.5, MPO:46.1 (all chronic)	**Stroke** Agrammatic aphasics, mixed levels of severity and aphasia type	**Yes No** treatment (N:6), and previously studied clinician-delivered treatment (N:8)	**Sentactics** TUF (treatment of underlying forms) delivered by virtual clinician	6–8 weeks, 4 1 h sessions per week or until criterion	EG showed more gains on trained items than CG, no difference with clinician delivered; EG showed gains on Cinderella narrative and OR^*^ sentences on NAVS^*^
	Linebarger et al., 2007	N:6, age:49.8, MPO:~35 (all chronic)	**Stroke** Non-fluent aphasia	**Yes** Healthy controls	**SentenceShaper** Records words or phrases in the patient's voice, represented with visual icons to replay and order into larger units; word-finding assistance	11–23 weeks; variable intensity decided by patient; average: 29.43 h total	Results varied; most showed some gains on trained narratives
	Crerar et al., 1996	N:14, age:52.4, MPO:~52 (all chronic)	**Stroke (mostly)** Mixed levels of severity and aphasia type (no global)	**No**	Picture-building and sentence-building modes in both verb and preposition treatment	6 weeks (3 weeks of each type of treatment), two 1 h sessions per week	Probe measures varied by patient regardless of treatment given first; treating verbs first showed clearer gains
Language, Single domain, Writing	Seron et al., 1980	N:5, age:42.8, MPO: ranged (all but one chronic)	**Stroke (mostly)** Mixed levels of severity	**No**	Keyboard writing to dictation with adaptive different levels of cueing	Ranged from 4 to 10 weeks, total sessions ranged from 7 to 30	Probe measures of words with at least one error and total number of errors decreased from pre to post testing
	Laganaro et al., 2006	N:8, age:49.6, MPO:1-2 (all acute)	**Stroke and TBI** Mixed levels of severity and aphasia type	**No**, alternating between treatment sets of differing lengths	Computerized written naming program with cues provided on either a set of 48 or 96 items	2 weeks, 5 days a week for 30–60 min	All but one patient improved in picture naming after treatment and this improvement was limited to the list that was trained at each assessment period
Language, Multiple domains	Choi et al., 2016	N:8, age:50.8, MPO:30 (most were chronic)	**Stroke** Mixed levels of severity and aphasia type	**No**	**iAphasia** Six domains of tasks with differing levels of difficulty, hints provided	4 weeks, variable intensity decided by patient, average: 30.3 h total	K-WAB^*^ AQ scores improved
	Stark and Warburton, 2016	N:10 (3 pilot), age:63.6, MPO:39.2 (all chronic)	**Stroke** Expressive aphasia with intact comprehension	**No**, crossed design comparing treatment to Bejeweled game app (except for pilot patients)	**Language Therapy** (Tactus Therapy Solutions) Reading, naming, comprehension, and writing tasks	8 weeks (4 of each), variable intensity decided by patient	Significant gains on CAT^*^, improved on narrative measures; ES greater than control period
	Steele et al., 2014	N:9, age:61.4, MPO:66.8 (all were chronic)	**Stroke** Mixed levels of severity and aphasia type	**No**	**Lingraphica TalkPath** Various exercises with varied difficulty and self-administered hints; remote treatment (group and individual) with WebEx and GoToMeeting	12 weeks, 1 remote group session per week, every 4th week: individual, variable intensity decided by patient for homework	CETI and NOMS^*^ showed gains on most items administered, one item and overall score of RIC-CCRSA^*^ showed gains
	Corwin et al., 2014	N:6, age:52, MPO:37 (all chronic)	Stroke (1 RH^*^) Mixed levels of severity and aphasia type	**No**, alternated between computer-based and no treatment (crossed design)	**Parrot software** Semantic feature, confrontation naming, and sentence completion tasks	4 weeks, 4 sessions of 2 h/week	BNT and WAB Naming and Word Finding subtests improved (trained words excluded)
	Mortley et al., 2004	N:7, age:61.7, MPO:~60 (all chronic)	**Stroke**	**No**	**StepByStep** Exercises involving word-to-picture matching, semantic association, naming, reading, and spelling	27 weeks, variable intensity decided by patient, average:2 h and 45 min per week	Gains in trained words for all pts
	Palmer et al., 2012	N:28 (33 at baseline), age:69.5 (EG), 66.2 (CG), MPO:74.4 (EG), 79.2 (CG) (all chronic)	**Stroke** Mixed levels of severity and aphasia type	**Yes** Usual care treatment (N:13)	**StepByStep** Self-managed computer word finding with cues	5 months, variable intensity decided by patient, average practice: 25 h total	EG showed more gains on naming in treatment than CG
Language and Cognitive	Des Roches et al., 2015	N:51, age:64.2, MPO:59.6 (most were chronic)	**Stroke (mostly)** Mixed levels of severity and aphasia type	**Yes** Only received treatment in clinic (N:9)	**Constant Therapy** 37 evidence-based cognitive and language tasks with different levels of difficulty; self-administered hints	10 weeks CG: 1 h/week, EG:1 h/week + homework: variable intensity decided by patient, average:4 h 8 min per week	Almost all patients showed gains on treatment tasks, EG more than control; EG showed more gains on standardized measures (WAB-R CQ^*^, AQ, several CLQT^*^ scores) than CG (PAPT^*^)
	Hoover and Carney, 2014	N:20 (3 cohorts), age:55–61, MPO:44–70 (depending on cohort) (all chronic)	**Stroke** Severity ranged from mild to moderate-severe	**No**	ICAP combined with multiple applications (Language Builder, SmallTalk, VASTtx, Language Therapy, and Constant Therapy)	4 weeks, 5 days/week for 6 h/day plus homework	Gains on narrative measures, several PALs^*^, PNT^*^, VNT^*^, F-A-S Word Fluency Task, DCT^*^, SIS^*^, and ALA^*^ or ASHA-FACS^*^
	Wcislo et al., 2010	N:63	**Stroke**, 33 with aphasia: mixed levels of severity and aphasia type, 30 with cognitive dysfunctions	**No**	Logopedic, physical, and cognitive exercises	3 months	Patients with aphasia showed 10 point change on comprehension test and 16 point change on verbal expression scores
	Wenke et al., 2014	N:39 (overlap in cohorts), age:60's, MPO:6–27.5 (depending on cohort)	**Stroke**, Varied severity	**Yes**, standardized service compared to three EGs: computer treatment (N:13), group treatment (N:11), and SPTA treatment (N:7)	Computer group used multiple online treatment programs: REACT-2, Aphasia Tutor, Language Links, and Synonyms, Homonyms, and Antonyms	8–10 weeks, 3–4 sessions per week on average for experimental groups	All groups showed gains on language production CAT subtests, all three EG groups showed gains on Disability questionnaire of CAT; no between group differences on either measure

* *MPO, Months past onset; Stroke, unless noted, stroke is in left hemisphere; WAB, Western Aphasia Battery; BNT, Boston Naming Test; BDAE, Boston Diagnostic Aphasia Examination; PNT, Philadelphia Naming Test; PRT, Philadelphia Repetition Test; PORT, Philadelphia Oral Reading Test; AQ, Aphasia Quotient; EG, Experimental (technology-based) group; CG, Control group; PICA, The Porch Index of Communicative Ability; ES, Effect Size; POWERS, Profile of word errors and retrieval in speech; WAB-R, Revised Western Aphasia Battery; CETI, Communicative Effectiveness Index; QCL, Quality of Communication Life Scale; CD, Communication Difficulty; BOSS, The Burden of Stroke Scale; OR, Object Relative; NAVS, Northwestern Assessment of Verbs and Sentences; CADL, Communication Activities of Daily Living; K-WAB, Korean version of the Western Aphasia Battery; CAT, Comprehensive Aphasia Test; NOMS, National Outcomes Measurement System; RIC-CCRSA, Rehabilitation Institute of Chicago—Communication Confidence Rating Scale for Aphasia; RH, Right Hemisphere; CQ, Cortical Quotient; CLQT, Cognitive Linguistic Quick Test; PAPT, Pyramids and Palm Trees; ICAP, Intensive comprehensive aphasia programs; PALs, Psycholinguistic Assessment of Language; VNT, Verb Naming Test; DCT, Discourse Comprehension Test; SIS, Stroke Impact Scale; ALA, Assessment of Living with Aphasia; ASHA-FACS, American Speech-Language-Hearing Association—Functional Assessment of Communication Skills for Adults*.

**Table 2:** Additional information about previous technology-based rehabilitation studies that have provided language rehabilitation to individuals with aphasia, separated by the domain(s) treated.

**Domain(s) treated**	**Study**	**Was the treatment tailored?**	**Home practice**	**Varied intensity**	**Within task improvement**	**Within task generalization**	**Maintenance**	**Impairment-based improvement**	**Functional/quality of life improvement**
Language, single domain, naming	Aftonomos et al., 1997	X	X	X				X	
	Fink et al., 2002	X	X (partial guidance group)		X	X	X	X	
	Raymer et al., 2006	X		X - compared two levels	X	X		X	
	Ramsberger and Marie, 2007	X	X	X - compared two levels	X	X	X		
	Doesborgh et al., 2004							X	
	Loverso et al., 1992				X			X	
	Bruce and Howard, 1987				X	X			
	Fridriksson et al., 2009		X		X			X	
	Harnish et al., 2014	X			X	X	X		
	Kurland et al., 2014	X	X	X	X			X	
	Woolf et al., 2016	X	X	X	X		X		
Language, single domain, reading	Katz and Wertz, 1992	X				X		X	
	Katz and Wertz, 1997	X						X	
	Cherney, 2010							X	
Language, single domain, sentence processing and production	Cherney and Halper, 2008	X	X	X	X		X	X	X
	Manheim et al., 2009	X	X	X			X		X
	Cherney et al., 2014	X	X		X	X	X		
	Kalinyak-Fliszar et al., 2015				X	X		X	
	Thompson et al., 2010				X			X	
	Linebarger et al., 2007	X	X	X				X	
	Crerar et al., 1996				X	X			
Language, single domain, writing	Seron et al., 1980	X		X		X	X		
	Laganaro et al., 2006			X - compared two levels of item numbers	X		X		
Language, multiple domains	Choi et al., 2016	X	X	X			X	X	
	Stark and Warburton, 2016	X	X	X			X	X	
	Steele et al., 2014	X	X	X					X
	Corwin et al., 2014							X	
	Mortley et al., 2004	X	X	X	X	X			
	Palmer et al., 2012	X	X	X	X		X		
Language and cognitive	Des Roches et al., 2015	X	X	X - compared EGs who receive more intensive than CG	X			X	
	Hoover and Carney, 2014	X	X					X	X
	Wcislo et al., 2010	X						X	
	Wenke et al., 2014	X		X - compared EGs who also receive more intensive than CG				X	X

*The table provides binary metrics about the design of the studies, including whether the study had tailored treatment, provided home practice, varied the level of intensity of treatment (denoted by an X if these aspects were included in the design of the study). Additionally, the table provides a binary metric about the outcomes that were utilized in the study (highlighted in gray if not used) and whether those outcomes showed quantitative improvement in at least one examined measure (denoted by an X if improvement was demonstrated)*.

### What have these previous studies examined?

Table 1 is organized in terms of the domains addressed and the types of treatments that were investigated and provides details about the methods and results. For the purpose of brevity, these studies are not described here in detail. Within the language domain, studies have typically provided a single domain intervention, such as naming, reading words or sentences and sentence production. As reviewed in Table 1, these studies include programs that target specific aspects of reading, naming or production.

#### Language rehabilitation in a single domain: naming

One program, called Lingraphica (Lingraphica, The Aphasia Company, Princeton, NJ), is a computer-delivered naming treatment that provides interactive lexical items that the participants can click on that provides the written and spoken name of the item, in a field of semantically related items. A study that investigated the effectiveness of Lingraphica (Aftonomos et al., 1997) with no other concurrent treatment found it elicited gains on all standardized measures that were administered [Western Aphasia Battery (WAB, Kertesz, 1982), Boston Naming Test (BNT, Goodglass et al., 1983), and Boston Diagnostic Aphasia Examination (BDAE, Kaplan, 1983)]. Another program called MossTalk (Moss Rehabilitation Research Institute, Elkins Park, Pennsylvania) is a computer delivered naming treatment that provides various levels of cues to facilitate naming or multimodal matching exercises to work on lexical comprehension. Three studies investigated the effectiveness of MossTalk (Fink et al., 2002; Raymer et al., 2006; Ramsberger and Marie, 2007) and found gains in all cohorts on performance within the program. Also, two of the studies showed limited gains on standardized measures [Philadelphia Naming Test (PNT, Roach et al., 1996), Philadelphia Repetition Test (PRT, Dell et al., 1997), Philadelphia Oral Reading Test (PORT, part of the PNT), WAB, and BNT]. Multicue is a program that provides self-cued naming treatment, which has been used by one study (Doesborgh et al., 2004) that found significant gains on the BNT. Finally, several studies have investigated the effectiveness of general naming treatment provided through computers or tablets (Bruce and Howard, 1987; Loverso et al., 1992; Fridriksson et al., 2009; Harnish et al., 2014; Kurland et al., 2014; Woolf et al., 2016), all of which found gains for performance on trained items and the studies that tested standardized measures showed corresponding improvement (PNT, BDAE, BNT, Porch Index of Communicative Ability (PICA, Porch, 1971); as well as other measures such as the profile of word errors and retrieval in speech (POWERS, Herbert et al., 2013).

#### Language rehabilitation in a single domain: reading

There are several studies that investigated computer-based reading treatments. Two studies (Katz and Wertz, 1992, 1997) provided a computer-based hierarchical reading program. One of the studies found improvements on items trained during the treatment program (Katz and Wertz, 1992) and both studies showed gains on the PICA and WAB scores. One study (Cherney, 2010) provided a computerized version of oral reading for language in aphasia (ORLA) treatment, a software program that involved systematic and repeated practice in reading aloud of sentences and paragraphs, which resulted in gains (similar to those when treated by clinician) on Revised WAB (WAB-R, Kertesz, 2007).

#### Language rehabilitation in a single domain: sentence processing and production

There are several programs that provide sentence processing and/or production treatments through the use of technology. The AphasiaScripts program (Rehabilitation Institute of Chicago, Chicago, IL) provides script training treatment with diminishing cues through a virtual therapist to help patients with their speech production skills in a guided context. Three studies investigated the effectiveness of AphasiaScripts (Cherney and Halper, 2008; Manheim et al., 2009; Cherney et al., 2014) and found gains for performance within the program and on standardized measures [WAB-R, Communicative Effectiveness Index (CETI, Lomas et al., 1989), Quality of Communication Life scale (QCL, Paul-Brown et al., 2003), and the Communication Difficulty subscale of the Burden of Stroke Scale (BOSS, Doyle et al., 2004)]. Another study (Kalinyak-Fliszar et al., 2015) investigated a different type of script training treatment to improve production and found improvement in production of the trained scripts and some improvement on discourse narratives.

Another program called Sentactics, is a computerized treatment of underlying forms (TUF) treatment, provided by a virtual clinician. The efficacy of this treatment method has been investigated in one study (Thompson et al., 2010), which found gains for performance within the program and on standardized measures [the Northwestern Assessment of Verbs and Sentences (NAVS) (Cho-Reyes and Thompson, 2012) and Cinderella narrative]. SentenceShaper (Psycholinguistic Technologies, Inc., Jenkintown, PA) is another software program which records patients' spoken words or phrases and allows them to reorder into sentences and narratives. One study that investigated the effectiveness of SentenceShaper (Linebarger et al., 2007) found gains in the practiced narratives. A different software program to promote word and sentence production by training verbs and prepositions in both a picture-building and a sentence-building mode (Crerar et al., 1996) found that patients trained on the program improved on production.

#### Language rehabilitation in a single domain: writing

Only two studies investigated the effectiveness of technology that treated only writing. One study (Seron et al., 1980) provided a writing treatment delivered with a computer which focused on typing to dictation and found improvement on a probe measure of writing to dictation. Another study (Laganaro et al., 2006) provided a computer-based writing treatment of picture spelling and found improvement on a probe measure of confrontation naming.

#### Language rehabilitation in multiple domains

While all the above studies provided treatment in a single domain of language, there are other programs that target multiple domains, including iAphasia, which is a program that provides treatment in six domains (auditory comprehension, reading comprehension, repetition, naming, writing, and reading). One study tested the effectiveness of iAphasia (Choi et al., 2016) and found scores improved on the Korean version of the WAB (K-WAB, Kim and Na, 2004). Another app called Language Therapy (Tactus Therapy Solutions Ltd., Vancouver, BC, Canada) comprises reading, naming, comprehension, and writing tasks. Researchers studying Language Therapy (Stark and Warburton, 2016) found gains on standardized measures [the Comprehensive Aphasia Test (CAT, Swinburn et al., 2004) and narrative measures]. An updated Lingraphica program, TalkPath, provides a variety of exercises focusing on listening, speaking, reading, and writing. One study (Steele et al., 2014) tested the effectiveness of the program along with remote group and individual treatment sessions and found improvement on several standardized measures [CETI, the National Outcomes Measurement System (NOMS, American Speech-Language-Hearing Association, 2003), and Rehabilitation Institute of Chicago's Communication Confidence Rating Scale for Aphasia (CCRSA-RIC, Babbitt et al., 2011)]. Parrot software (Parrot Software, West Bloomfield, MI) is a program that provides tasks that focus on semantic features, confrontation, and sentence completion. One study (Corwin et al., 2014) investigated the effectiveness of the program and found gains on standardized measures that tested naming (BNT and WAB-R naming subtests). Finally, StepByStep (Steps Consulting Ltd., Hauz Khas, New Delhi, India) is a self-managed computer word finding program and exercises involving word-to-picture matching, semantic associates matching, reading, and spelling. StepByStep has been utilized in two studies (Mortley et al., 2004; Palmer et al., 2012), both of which found gains in performance within the program.

#### Language and cognitive rehabilitation

A few studies have combined the delivery of language and cognitive rehabilitation exercises. These studies provide a broader range of therapy exercises and investigate the interaction between language and cognition in terms of whether there are improvements across these domains. One such program is Constant Therapy (iTherapy, Constant Therapy Inc., Newton, MA), which provides several domains of language and cognitive tasks including naming, reading, writing, sentence completion, visuo-spatial processing, memory, attention, problem solving, and executive function (Kiran et al., 2014). One study tested the effectiveness of Constant Therapy (Des Roches et al., 2015) and found improvement on both performance within the program and standardized measures. Another study (Hoover and Carney, 2014) looked at the implementation of tablet-based treatment programs in an intensive, comprehensive aphasia program (ICAP), including Constant Therapy, Language Builder (Mobile Education Store, LLC, Salem, OR) which is a program that provides practice for sentence production with photographic stimulus, SmallTalk (Lingraphicare, Inc., Princeton, NJ) which is a series of applications that include functional phrases that can be used for communication and/or for speech practice, and VASTtx (Speak in Motion, LLC, Vienna, VA), a program that provides visual models for the production of phonemes, keywords, and customizable playlists, and Language Therapy. Hoover and Carney found improvements on several standardized measures [Psycholinguistic Assessment of Language (PALs), PNT, Northwestern Verb Naming Test (VNT, part of the Verb Production Battery), F-A-S Word Fluency Task, Discourse Comprehension Test (DCT, Brookshire and Nicholas, 1993), Stroke Impact Scale (SIS, Duncan et al., 1999), and Assessment of Living with Aphasia (ALA, Kagan et al., 2010), or American Speech-Language-Hearing Association-Functional Assessment of Communications Skills for Adults (ASHA-FACS, American Speech-Language-Hearing Association, 1995)]. One study (Wcislo et al., 2010) investigated the effectiveness of using several logopedic, physical, and cognitive exercises delivered through a computer over the internet and found gains on standardized measures. Finally, one study (Wenke et al., 2014) tested the effectiveness of using several computer-based programs including REACT-2, React2 Ltd., (Peebles, UK, which works on auditory processing, visual processing, semantics, memory, and life skills), Aphasia Tutor (Bungalow Software, Blacksburg, VA), which works on speech, word-retrieval, reading, and writing; Language Links; and Synonyms, Homonyms, and Antonyms) and found improvement on the CAT.

While all of these studies appear to find improvements on at least one of the measures examined, there is a great deal of variability across the studies in terms of methods, design, and results. Nonetheless, these studies comprise an important body of research that can provide insights into our understanding of the nature of technology-based rehabilitation approaches.

## Questions that can now be examined

**Figure 2:**
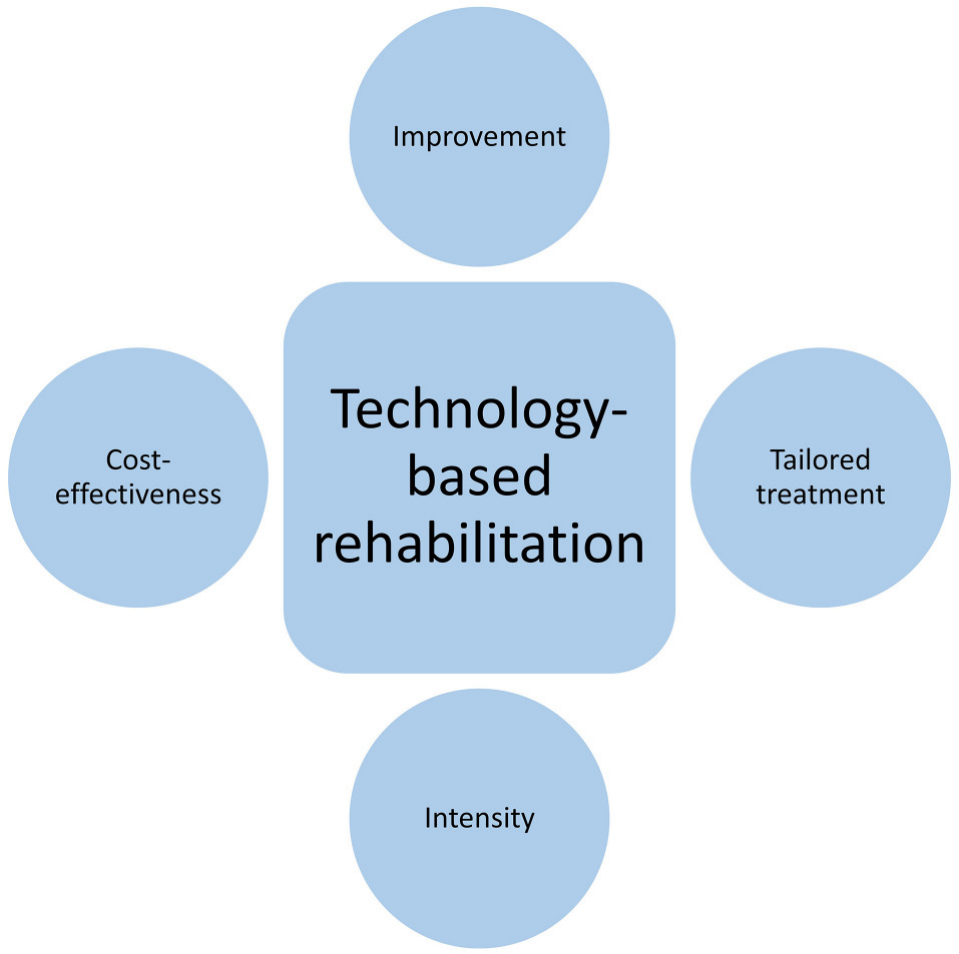
Schematic depicting the different factors examined across the previous studies, namely improvement on various outcome measures, tailored treatment, intensity of the rehabilitation, and cost-effectiveness.

The previous studies reviewed provide a basis for the effectiveness of technology-based rehabilitation for individuals with acquired brain injury/stroke and allows for further, more specific questions to be addressed. Some of these questions address the viability and utility of a technology-based treatment delivery mechanism, such as whether a **treatment can be standardized** and whether it includes **homework practice**. Other questions evaluate the effectiveness of rehabilitation in general, including the intensity of rehabilitation provided and how improvement is captured across different outcome measures. Figure 2 provides a breakdown of these factors and Table 2 identifies which of the studies referenced in Table 1 incorporate these aspects.

KEY CONCEPT 3 **Standardized treatment**

Refers to how treatment is delivered to individuals. This method delivers treatment the exact same way to all individuals, i.e., the treatment is standardized across individuals.

KEY CONCEPT 4 **Homework practice**

Refers to treatment at home, which is beneficial because it allows individuals to practice the treatment sessions outside of the clinic. This increases the overall practice that patients can potentially obtain and capitalizes on one of the main advantages of technology-based treatment, which is ease of accessibility.

### Improvement

To fully understand what the improvements observed across the various studies mean, it is important to provide an appropriate context. One way to look at improvement is to see whether the specific tasks delivered by the programs show improvement and to what extent performance on those tasks/sets of stimuli help improve performance on other tasks/sets (i.e., generalization). Another way to examine improvement is to see whether training on the tasks results in improvement on standardized tests. Different studies have looked at different measures on standardized tests, therefore, is it useful to classify the difference in improvement based on the type of measures that are utilized (i.e., impairment or functional/quality of life (QOL) measures). Figure 1 shows a schematic of how these outcomes relate to the targeted treatment. At the first level, treatment is expected to improve performance on the task trained, at the next level, this is expected to generalize to untrained items or other tasks. Next, the treatment might influence individuals' scores on impairment-based standardized measures, and followed by scores on functional and QOL measures. This progression illustrates if and how a particular treatment can have wide ranging impact on one's overall communication and QOL. Another factor worth considering is if the treatment has a long lasting impact on an individual's behavior (on either within task improvement or on standardized measures). This can be assessed in terms of whether studies conducted follow-up examinations (maintenance) and found the improvements to maintain over time on either within task improvements or on standardized measures (meaning that the standardized measures were administered multiple times and the gain found after treatment was sustained).

Of the 11 studies that focused treatment on only the naming domain (Bruce and Howard, 1987; Loverso et al., 1992; Aftonomos et al., 1997; Fink et al., 2002; Doesborgh et al., 2004; Raymer et al., 2006; Ramsberger and Marie, 2007; Fridriksson et al., 2009; Harnish et al., 2014; Kurland et al., 2014; Woolf et al., 2016), nine tested and found within task improvement, five of six studies that examined within task generalization found improvement, seven of eight studies that looked for gains on impairment-based measures found improvement, and the one study that examined functional/QOL measures did not find improvement. Additionally, four studies tested and found maintenance of within task improvement or maintenance of treatment-induced gains on standardized measures. Of the three studies that focused treatment on only the reading domain (Katz and Wertz, 1992, 1997; Cherney, 2010), none looked for within task improvement, one study examined and found within task generalization, all three tested and found gains on impairment-based measures, while none looked for functional/QOL measurement improvement. None of the studies tested maintenance of within task improvement or maintenance of treatment-induced gains on standardized measures.

Of the seven studies that focused treatment on only sentence processing or production (Crerar et al., 1996; Linebarger et al., 2007; Cherney and Halper, 2008; Manheim et al., 2009; Thompson et al., 2010; Cherney et al., 2014; Kalinyak-Fliszar et al., 2015), five studies tested and found within task improvement, three studies looked for and found within task generalization, four studies examined and found gains on impairment-based measures while two studies tested and found functional/QOL measurement improvement. Three studies looked for and found maintenance of within task improvement or maintenance of treatment-induced gains on standardized measures. Of, the two studies that focused treatment on the writing domain (Seron et al., 1980; Laganaro et al., 2006), one study examined and found within task improvement while the other tested and found within task generalization. Both studies looked for and found maintenance of that improvement.

Of the six studies that focused on multiple language domains (Mortley et al., 2004; Palmer et al., 2012; Corwin et al., 2014; Steele et al., 2014; Choi et al., 2016; Stark and Warburton, 2016), two studies examined and found within task improvement, one study tested and found within task generalization, three of four studies that looked for gains on impairment-based measures found improvement, while one study examined and found functional/QOL measurement improvement. Three studies tested and found maintenance of within task improvement or maintenance of treatment-induced gains on standardized measures. Of the four studies that focused on both language and cognitive domains (Wcislo et al., 2010; Hoover and Carney, 2014; Wenke et al., 2014; Des Roches et al., 2015), one study looked for and found within task improvement, none examined within task generalization, all four studies tested and found gains on impairment-based measures, while two studies found functional/QOL measurement improvement. None of these studies looked for maintenance of within task improvement or maintenance of treatment-induced gains on standardized measures.

To summarize, all the studies that have examined within task improvement have observed it, only one study did not find within task-generalization (Kurland et al., 2014). All but two studies (Steele et al., 2014; Woolf et al., 2016) found concurrent improvement on impairment-based measures. Fewer studies tested functional/QOL measures, and all but one study (Doesborgh et al., 2004) reported positive gains. Therefore, while there is a great deal of variability in the methods, design, and measures tested, the studies consistently demonstrated improvement and various degrees of generalization have been shown. Generalization can be considered a beneficial effect of treatment since it demonstrates efficiency for showing improvements beyond what was directly targeted. However, generalization can also be an issue when determining whether treatment was the reason for any improvement (Figure 1), which highlights the need for further research systematically examining each level of improvement across various rehabilitation programs.

### Is rehabilitation tailored for patients?

One of the advantages of a computer-based rehabilitation is that it allows researchers to deliver rehabilitation the exact same way to all individuals, i.e., standardizing the delivery of rehabilitation. Such an approach could potentially deviate from the traditional ways of rehabilitation, where clinicians **tailor their treatment** for individuals. However, in traditional rehabilitation approaches, even though clinicians tailor the treatment, such rehabilitation is hard to replicate across patients. In this context, technology can provide the best of both worlds; technology can provide rehabilitation in a standardized manner and allows a clinician or researcher to tailor the tasks and items to an individual's deficits. Reviewing these studies allows us to examine to what extent studies deliver standardized treatment or tailor the treatment program for individual patients. For instance, ten of the studies (Bruce and Howard, 1987; Loverso et al., 1992; Crerar et al., 1996; Doesborgh et al., 2004; Laganaro et al., 2006; Fridriksson et al., 2009; Cherney, 2010; Thompson et al., 2010; Corwin et al., 2014; Kalinyak-Fliszar et al., 2015) required all participants to proceed through the same set of items and/or tasks. Therefore, rehabilitation in these studies was not tailored to individual patients.

KEY CONCEPT 5 **Tailored treatment**

Refers to how treatment is delivered to individuals. This method delivers treatment that is specific to an individual's deficits and is therefore tailored to the individual.

Kurland et al. (2014) noted that their participants may have benefited from an increase in task difficulty after demonstrating improvement. This finding suggests that rehabilitation may not be best provided in a one-size-fits all manner, even if the item sets are tailored. Instead, rehabilitation should be dynamically adapted to the individual in terms of difficulty level, yet still be provided in a standardized way. Computer programs designed with algorithms allow for this manner of rehabilitation to be possible. Apart from the studies mentioned above, other studies in this review provided more individualized rehabilitation in terms of tailoring the item sets trained for each patient or by either allowing the clinician or researcher to tailor the tasks or the patients to choose which tasks to work on. For both standardized and tailored studies, the outcomes demonstrated generally positive results, however, it appears that individualizing treatment results in greater overall gains across the range of outcome measures, whereas studies that provided standardized treatment only measured and found gains in the within task measure, generalization, or impairment-based measures.

### Homework practice

An important component of computer-based treatments is to allow for patients to practice the treatment outside of the clinic as part of their homework. This increases the amount of overall practice that patients can obtain and capitalizes on the ease of accessibility, which is another advantage of computer based-treatment. Several of the studies reviewed provided specific instructions for participants to practice treatment tasks at home. Seventeen studies (Aftonomos et al., 1997; Fink et al., 2002; Mortley et al., 2004; Linebarger et al., 2007; Ramsberger and Marie, 2007; Cherney and Halper, 2008; Fridriksson et al., 2009; Manheim et al., 2009; Palmer et al., 2012; Cherney et al., 2014; Hoover and Carney, 2014; Kurland et al., 2014; Steele et al., 2014; Des Roches et al., 2015; Choi et al., 2016; Stark and Warburton, 2016; Woolf et al., 2016) prescribed homework in addition to or instead of the treatment sessions. The remaining studies (Seron et al., 1980; Bruce and Howard, 1987; Katz and Wertz, 1992, 1997; Loverso et al., 1992; Crerar et al., 1996; Doesborgh et al., 2004; Laganaro et al., 2006; Raymer et al., 2006; Cherney, 2010; Thompson et al., 2010; Wcislo et al., 2010; Corwin et al., 2014; Harnish et al., 2014; Wenke et al., 2014; Kalinyak-Fliszar et al., 2015) only provided the computer-based treatment in the clinic when patients made their periodic visits.

To summarize, while studies that provide treatment only in the clinic show gains in outcomes, studies that provide homework are also quite effective. Only one study, Des Roches et al. (2015), compared patients who received homework to those that did not and found that the experimental patients (who received homework in addition to clinic time) improved on a greater number of tasks within the program and on a greater number of impairment-based measures. Future studies will need to examine these types of comparisons to ascertain whether providing homework treatment is as effective as traditional treatment and if there are any benefits to costs savings with home-based treatment.

### Intensity

Another potential advantage of computer-based rehabilitation is that, if indeed patients can easily access the prescribed rehabilitation wherever they have access to a computer, patients could potentially practice treatment more often. The question of whether increasing the intensity of rehabilitation is beneficial to outcomes has been a focus of traditional rehabilitation studies. While it is logical to assume that more intensive treatment results in greater outcomes and has been demonstrated in chronic (Bhogal et al., 2003a,b; Cherney et al., 2008) and in acute patients with aphasia (Godecke et al., 2014); other studies have questioned this premise (Bakheit et al., 2007; Dignam et al., 2015). For instance, a randomized controlled trial found that intensive treatment (up to 5 h/week) was no better than standard treatment (1–2 h/week) (Bakheit et al., 2007). Therefore, it is currently difficult to draw conclusions about what the optimal intensity and duration of rehabilitation should be for individual patients with aphasia.

Two studies (Raymer et al., 2006; Ramsberger and Marie, 2007) specifically varied the intensity of treatment. These studies found that all patients showed gains on within task performance and generalization to untrained items during both lower and higher intensity phases. Raymer et al. (2006) found greater gains in the higher intensity phase and, however, minimal improvement in impairment-based measures. Ramsberger and Marie (2007) found maintenance of the within task improvement. One study (Laganaro et al., 2006) also examined the effect of number of items treated as a level of intensity using a crossed design method, which found that, while the proportion of improvement was similar for both the smaller and larger (double the number of items) sets, there was a significantly greater improvement on the larger set, even though these items received less exposure in the treatment. This shows that exposure to a greater number of items provides proportionally greater amount of improvement than if exposed to a smaller number of items. Therefore, the higher number of repetitions in the smaller set did not result in greater improvement. Though this result does not contradict the effects of intensity in the previously mentioned studies, it might highlight the importance of greater intensity providing not only more repetitions for items but also exposure to a higher number of items.

Several of the studies reviewed prescribe a certain amount of home practice and in some of these studies, the homework was used to supplement treatment already given in the clinic. Twelve studies (Aftonomos et al., 1997; Mortley et al., 2004; Linebarger et al., 2007; Cherney and Halper, 2008; Manheim et al., 2009; Palmer et al., 2012; Kurland et al., 2014; Steele et al., 2014; Des Roches et al., 2015; Choi et al., 2016; Stark and Warburton, 2016; Woolf et al., 2016) prescribed homework to the patients but did not prescribe the amount/intensity of homework practice (instead suggesting a target time of practice). Of these studies, four used the homework to supplement clinic work (Aftonomos et al., 1997; Steele et al., 2014; Des Roches et al., 2015; Woolf et al., 2016), which potentially provided greater intensity of practice for the patients. The remaining eight studies used the homework as the only means of the therapy; some of the studies asked participants to practice for 20–30 min per day or on a specific number of days. If participants followed the prescribed amount of homework, they would not have received a greater intensity of therapy than if they came into the clinic, although one study (Choi et al., 2016) asked patients to practice at home for as often and as long as possible and found their participants received, on average, seven and a half hours of treatment per week. When examining treatment time across these twelve studies, home practice was not always documented but when it was, patients practiced for roughly four and a half hours on average [across the studies that report average usage (Aftonomos et al., 1997; Mortley et al., 2004; Linebarger et al., 2007; Manheim et al., 2009; Palmer et al., 2012; Kurland et al., 2014; Des Roches et al., 2015; Choi et al., 2016)]. These studies show that homework may be a way to increase the amount of practice that patients receive.

Additionally, in two studies, the control groups had inherently lesser frequency of therapy practice than the experimental groups. Des Roches et al. (2015) found that the experimental patients, who received homework in addition to clinic time, improved on a greater number of tasks within the program and on a greater number of impairment-based measures. Wenke et al. (2014) found that, while both the control group and the experimental groups showed gains on impairment-based measures, only the experimental groups showed gains on the functional/QOL measure. Taken together, these studies imply that greater intensity positively impacts treatment outcomes across the range of outcomes (Figure 1) that were examined.

Apart from the above mentioned studies, the other studies included in this review prescribed an amount of treatment time or criterion of performance to reach (while keeping intensity consistent). While the results from studies that prescribed homework and those that did not were both generally positive, one study (Choi et al., 2016) which allowed patients to decide the intensity of their homework practice found a positive relationship between the amount of practice with the program and scores on the K-WAB, where the participants who had a higher intensity of treatment showed greater improvement. Future studies should examine the relationship between amount of practice and treatment outcomes so that it can determined whether individually varied practice time can influence overall treatment outcomes.

### Cost-effectiveness

Although only a few studies have tested the cost-effectiveness of technology-based rehabilitation compared to standard face-to-face rehabilitation in individuals with acquired brain injury/stroke, the studies that have examined this factor have found it to be a cost effective alternative. Palmer et al. (2012) calculated the cost of using the StepByStep program compared to the cost of usual care and estimated that the use of technology increased the cost over an individual's lifetime by a small amount. However, when considering the additional gains the experimental group made compared to the control group, the technology-based rehabilitation is considered to be quite cost-effective. Wenke et al. (2014) calculated the cost of standard service compared to three more intensive service models: computer treatment, group treatment, and treatment with a speech pathology therapy assistant (SPTA), and found the total costs were lowest for standard service and highest for SPTA treatment, with computer and group treatments being equal. However, once the cost of treatment per hour per client was calculated, the computer and group treatment models were the least costly, followed by standard service and then SPTA treatment. This is an area of further need of research if technological-based rehabilitation approaches have a long term future in the delivery of clinical services.

## The ultimate goal: personalized rehabilitation that provides independence

In summary, this review details the various studies that have examined technology-based rehabilitation for individuals with brain injury. In contrast to therapist-based rehabilitation, technology can provide individuals with aphasia access to home practice and therefore greater intensity in a cost-effective manner (Figure 2). While there exists quite a bit of data on the effectiveness for demonstrating various degrees of improvement (see Figure 1), future research needs to continue to systematically study the effect of treatment on generalization, the effect of homework and intensity on improvement, and the cost-effectiveness of technology-based rehabilitation (Figure 2). Ultimately, most individuals with acquired brain injury who require rehabilitation services live with a chronic disability. The ideal rehabilitation program provides a **personalized rehabilitation** plan that offers a step in the journey toward greater independence by empowering the individual toward being more engaged and integrated in their own care. The integration of technology-based rehabilitation may allow this goal to become a reality for individuals with acquired brain injury, however further research is still necessary. Additionally, integrating impairment-based rehabilitation tasks with functional uses of technology elicits broader improvement and allows the patient to achieve greater independence. Several reviews and commentaries have previously explored this capacity of technology (van de Sandt-Koenderman, 2004, 2011; Brandenburg et al., 2013; Hoover and Carney, 2014; Ramsberger and Messamer, 2014; Szabo and Dittelman, 2014). This integrated approach may provide greater functional gains and improved QOL than face-to-face rehabilitation alone. Therefore, technology-based rehabilitation should not replace rehabilitation with a clinician, but should instead be a supplemental tool used both in the clinic and at home. Another benefit of technology-based rehabilitation programs is the ability to collect large amounts of data. This could allow for the creation of decision support tools, which could facilitate optimized, patient-centered, and evidence-based decisions. The use of technology-based rehabilitation can further the field of evidence for the factors discussed above and can allow clinicians to make the most clinically informed decisions about their patients' rehabilitation plans.

KEY CONCEPT 6 **Personalized rehabilitation**

Refers to what the ultimate goal of rehabilitation programs should be. This is achieved combining standardized and tailored treatment along with greater intensity of treatment (through homework practice). The further development of technology-based rehabilitation will allow this goal to become a reality for individuals with acquired brain injury.

## Author contributions

CD contributed to the acquisition and interpretation of the data for the work and contributed greatly to drafting and revising the work. SK contributed to the concept, design of, and interpretation of data for the work, and also contributed to revising the work.

### Conflict of interest statement

There is a significant financial relationship. Boston University owns a portion of stock equity in Constant Therapy, the software company that delivers the therapy. CD owns a portion of the stock equity that BU owns. SK is the co-founder and Scientific Advisor in Constant Therapy and owns stock equity in Constant Therapy. The results of the study are independent of the software platform and therefore there is no scientific overlap.

## Footnotes

^1^Studies with one or two participants were excluded due to the large amounts of single case studies. Interpreting the results from single case studies is difficult when also examining the results from studies with a larger number of participants.
